# Editorial: Advances in multi-omics technologies in pathophysiological processes and disease diagnostics

**DOI:** 10.3389/fcell.2025.1693388

**Published:** 2025-10-01

**Authors:** Xinru Liu, Wenwu Liu, Shikai Yu, Si Wu

**Affiliations:** ^1^ Shanghai University, Shanghai, China; ^2^ Navy Medical Unvierisity, Shanghai, China; ^3^ Shanghai Tenth People’s Hospital, Tongji University School of Medicine, Shanghai, China; ^4^ Computational Biology, Precision Medicine, Amgen, CA, United States

**Keywords:** multi-omics integration, biomarkers, machine learning, pathophysiological processes, precision medicine

## Introduction

The integration of multi-omics technologies has ushered in a transformative era for biomedical research, enabling a systems-level exploration of complex pathophysiological processes. By moving beyond the limitations of single-omics approaches, concurrent analysis of genomic, transcriptomic, proteomic, and metabolomic data provides a holistic view of disease mechanisms, revealing intricate molecular networks and dynamic interactions. This paradigm shift is critically advancing our capabilities in early disease detection, prognostic stratification, and the discovery of novel therapeutic targets, thereby paving the way for precision medicine.

This Research Topic, *Advances in Multi-Omics Technologies in Pathophysiological Processes and Disease Diagnostics*, presents five pioneering contributions that exemplify how integrative omics, combined with advanced computational tools, can address unmet clinical needs across a spectrum of human diseases—from neonatal disorders to cardiovascular and neurological conditions.

## Metabolomics and neonatal sepsis

Bian et al. applied metabolomics coupled with machine learning to tackle the challenge of early neonatal sepsis diagnosis (Bian et al.). Their work identified specific metabolic biomarkers with strong discriminatory power, providing a promising non-invasive strategy for timely and accurate diagnosis in this vulnerable population.

## Lactylation in CNS disorders

Tian et al. provided a comprehensive review of lactylation, a novel post-translational modification, and its implications in central nervous system disorders (Tian et al.). By linking cellular metabolism to epigenetic regulation, this work illustrates how multi-omics frameworks are essential for unraveling mechanistic connections between metabolic rewiring and neuronal function.

## Cardiotoxicity in cancer therapy

Ding et al. focused on doxorubicin-induced cardiotoxicity, a major hurdle in oncology, by performing metabolomic profiling (Ding et al.). Their findings identified plasma metabolite signatures that serve as sensitive early indicators of cardiac damage, offering opportunities for patient monitoring and the development of cardioprotective interventions.

## Mitochondrial biomarkers in ischemic stroke

Zhang et al. integrated bulk and single-cell RNA sequencing with machine learning to identify biomarkers associated with the mitochondrial unfolded protein response in ischemic stroke (Zhang et al.). This multi-layered approach revealed cell-type-specific contributions to stroke pathology and validated mitochondrial stress responses as potential therapeutic targets.

## Lipid metabolism in myocardial infarction

Chen et al. explored plasma lipidomic profiles in patients with acute myocardial infarction of different coronary occlusion types (Chen et al.). Their study uncovered distinct lipid signatures that provide insights into disease heterogeneity and could inform more precise diagnostic and risk stratification strategies.

## Concluding remarks

Together, these contributions underscore a common message: the integration of multiple omics layers, enhanced by computational modeling and machine learning, is indispensable for deconvoluting the complexity of human diseases. From neonatal sepsis to myocardial infarction, and from epigenetic regulation in the brain to cardiotoxicity and stroke, these studies demonstrate the capacity of multi-omics to uncover novel mechanisms, identify robust biomarkers, and reveal therapeutic opportunities not visible to single-level analyses.

At the same time, challenges remain, including the standardization of omics protocols, the development of robust integration pipelines, and the need for validation in large and diverse cohorts. Addressing these hurdles will be critical to translating multi-omics discoveries into clinical applications.

We extend our sincere gratitude to all the authors and reviewers for their invaluable contributions. We hope this Research Topic will inspire further interdisciplinary collaboration and accelerate the translation of multi-omics science into improved diagnostics and personalized medicine.

